# Effect of Increasing Total Solids Contents on Anaerobic Digestion of Food Waste under Mesophilic Conditions: Performance and Microbial Characteristics Analysis

**DOI:** 10.1371/journal.pone.0102548

**Published:** 2014-07-22

**Authors:** Jing Yi, Bin Dong, Jingwei Jin, Xiaohu Dai

**Affiliations:** National Engineering Research Center for Urban Pollution Control, College of Environmental Science and Engineering, Tongji University, Shanghai, People's Republic of China; Oak Ridge National Laboratory, United States of America

## Abstract

The total solids content of feedstocks affects the performances of anaerobic digestion and the change of total solids content will lead the change of microbial morphology in systems. In order to increase the efficiency of anaerobic digestion, it is necessary to understand the role of the total solids content on the behavior of the microbial communities involved in anaerobic digestion of organic matter from wet to dry technology. The performances of mesophilic anaerobic digestion of food waste with different total solids contents from 5% to 20% were compared and the microbial communities in reactors were investigated using 454 pyrosequencing technology. Three stable anaerobic digestion processes were achieved for food waste biodegradation and methane generation. Better performances mainly including volatile solids reduction and methane yield were obtained in the reactors with higher total solids content. Pyrosequencing results revealed significant shifts in bacterial community with increasing total solids contents. The proportion of phylum *Chloroflexi* decreased obviously with increasing total solids contents while other functional bacteria showed increasing trend. *Methanosarcina* absolutely dominated in archaeal communities in three reactors and the relative abundance of this group showed increasing trend with increasing total solids contents. These results revealed the effects of the total solids content on the performance parameters and the behavior of the microbial communities involved in the anaerobic digestion of food waste from wet to dry technologies.

## Introduction

Food waste (FW), usually from residential, commercial establishments, institutional and industrial sources, is generated at an ever-increasing rate (higher than 10% every year) with the rapid population growth and rising living standards in China [1]. It seems to be a good idea to reuse this favorable feedstock for energy recovery and municipal solid waste (MSW) reduction because FW contains high moisture and biodegradable organics and accounts for 40–50% of the weight of MSW. Anaerobic digestion (AD) is the most attractive and cost-effective technology for treating sorted organic fraction of MSW, especially food wastes [2]. Various AD processes have been widely developed in many countries for the treatment of FW.

So far, three main types of AD technologies have been developed according to the total solids (TS) content of feedstocks: conventional wet (≦10% TS), semi-dry (10–20% TS) and modern dry (≧20% TS) processes. Dry anaerobic digestion, so called “high-solids” technology, has become attractive and was applied widely because it requires smaller reactor volume, lower energy requirements for heating, less material handling, and so on [2]–[4]. The TS content of solid waste influences anaerobic digestion performance, especially biogas and methane production efficiency [5]. Previous reports have investigated that role of TS content on AD performance in order to determine conditions for optimum gas production. Abbassi-Guendouz et al., showed that the total methane production decreased with TS contents increasing from 10% to 25% in batch anaerobic digestion of cardboard under mesophilic conditions [6]. The results obtained by Duan et al., showed that high-solids system could reach much higher volumetric methane production rate compared with low-solids system at the same solid retention time (SRT) in mesophilic anaerobic reactors treating sewage sludge [3]. Forster-Carneiro et al., showed that the biogas and methane production decreased with the total solids contents increasing from 20% to 30% in dry batch anaerobic digestion of food waste [2].

Anaerobic digestion is a multi-stage biochemical process in which the complex organic materials undergo hydrolysis, acidogenesis, and methanogenesis in series and each metabolic stage is functioned by different types of microorganisms [4]. They are present in a mixed culture but differ in their nutritional and pH requirement, growth kinetics, and their ability to tolerate environment stresses [7]. Characterization of microbial community structures in anaerobic digesters has been attractive from the point of review of engineering because understanding of microbial behavior can provides valuable information to optimize fermentation process to favor efficient breakdown of wastes [8]. However, the available literature is mainly about performance and corresponding the structure and dynamic of microbial community in either thermophilic or mesophilic anaerobic digestion of food waste, or only simply about performance comparisons. The AD performances at steady state and the comprehensive characterizations of microbial community in anaerobic digestion of FW with different TS contents (wet, semi-dry and dry) were not compared in parallel. In order to increase the efficiency of anaerobic digestion of FW, it is necessary to understand the role of the TS contents on the behavior of the microbial community structure involved in the anaerobic digestion of degradation from wet to dry technology.

Recently, various molecular microbial ecology tools have been applied in numerous studies to analyze microbial communities in different anaerobic digesters and their influences on the efficiency and stability of AD processes [9]. Pyrosequencing, as a next generation sequencing technology, has gained increasing attention as a novel tool for studying the microbial diversity [4]. Recently, this technology has been widely and successfully used to characterize the microbial community structures in various environmental samples, such as source waster [10], membrane filtration systems [11], soil [12]. Meanwhile, the microbial community structures were compared by this technology in anaerobic digestion of food waste at different organic loading rates (OLRs) [4].

Hence, the aim of this study was to conduct a comprehensive comparison of the microbial community structure using 454 high throughput pyrosequencing technology and related these microbial findings to their respective performances of mesophilic anaerobic digesters treating FW with different TS contents ranging from 5%–20%. It was expected that the reported work herein will reveal the role of the TS content on the behavior of the microbial community structure to increasing TS contents and hence to effective guide high solids anaerobic digestion of FW and to optimize the operational conditions for high anaerobic digestion efficiency.

## Materials and Methods

### Substrates and inoculums

FW used in this study was collected every 30 days from a dining room at Tongji University in Shanghai. After removing bones, shells, and other indigestible materials, the FW was finely smashed using an electrical crusher and sufficient mixed and stored at 4°C. The TS of the FW ranged from 26% to 28% (w/w) and volatile solid (VS) accounted for 92%–95% of TS. The mesophilic seed sludge was obtained from a full-scale anaerobic digester at Bailonggang municipal wastewater treatment plant (WWTP) (Shanghai, China). It had TS of 4.1% (w/w) and VS of 52.3% of TS. The main characteristics (average data plus standard deviations in duplicate tests) of substrates and inoculums are listed in Table 1. The collected FW was heated to 35°C before daily feeding.

**Table 1:** Characteristics of the substrates and inoculums.

Parameters	FW^a^ 1 (days 1–30)	FW 2 (days 31–60)	FW 3 (days 61–90)	FW 4 (days 91–120)	Inoculums
TS^b^ (%, w/w)	26.5±0.6	27.8±1.1	27.3±1.2	26.8±1.2	4.1±0.1
VS^c^/TS (%)	94.7±3.9	92.2±3.7	93.4±4.6	93.9±4.2	52.3±2.4
pH	4.72±0.21	4.64±0.11	4.79±0.24	4.87±0.23	7.9±0.3
C/N (w/w)	13.4±0.6	14.2±0.7	13.9±0.4	13.6±0.6	-
TAN^d^ (mg/L)	538±24	546±19	534±25	543±19	299±13

–Not determined.

a FW: food waste.

b TS: total solids.

c VS: volatile solids.

d TAN: total ammonia nitrogen.

### Reactors and operation

Three identical reactors (numbered R1, R1 and R3), with liquid working volume of 6.0 L, were equipped with helix-type stirrers to provide sufficient mixing for substrates. The rotation speed was set at a rate of 60 rpm (rotations per minute) with 9 min stirring and 1 min break, continuously. Daily feeding was carried out by pushing semi-fluid substrate through the feeding piston. Since the digestate of FW in each reactor was completely fluid, daily draw-off was easily carried out by opening the discharge valve.

On the first day of the experiments, 6.0 L seed sludge was added to each reactor, which was operated semi-continuously (once-a-day draw-off and feeding) under single phase mesophilic conditions (35°C). The reactors were purged with N_2_ for 10 min in order to provide anaerobic conditions. During the start-up period, the OLR was increased stepwise with high-solids FW before the TS content of the substrate in each reactor did not reach its designed TS level. Once the TS of the substrate in each reactor approached its designed level, the feeding FW was diluted to its designed TS level (5%, 15% and 20%, respectively) with de-ionized water before feeding. Each reactor was operated for five SRTs at 20 days SRT. For a full understading of the microbial community structures in anaerobic fermentation reactors with different TS contents, the anaerobically digested FW samples were taken on Day 100 when the systems could be deemed to have reached their steady state operation (determined by constant methane yield and VS reduction) after running for more than 3 months. The fermentation substrate samples in the reactors were taken every three days during the operation period of the fifth SRT for reactor performance analysis.

### DNA extraction, PCR and Pyrosequencing

To analyze the bacterial and archaeal communities in mesophilic anaerobic digesters with different feeding TS levels, 0.5 g of sample in reactor operated for 100 d was used for DNA extraction using a Fast DNA Spin Kit (QBIOgene, Carlsbad, CA, USA) following the manufacturer's instructions. For each sample, two independent PCR reactions were conducted using the primer pairs of 27F (5′-AGAGTTTGATCCTGGCTCAG-3′) and 533R (5′-TTACCGCGGCTGCTGGCAC-3′) for bacteria and 344F (5′-ACGGGGYGCAGCAGGCGCGA-3′) and 915R (5′-GTGCTCCCCCGCCAATTCCT-3′) for archaea [4]. To achieve the sample multiplexing during pyrosequencing, barcodes were incorporated in the 5′end of reverse primers 553R and 915R. All PCR reactions were carried out in a 25 uL mixture containing 0.5 uL of each primer at 30 mmolL^−1^, 1.5 uL of template DNA (10 ng), and 22.5 uL of Platinum PCR SuperMix (Invitrogen, Shanghai, China). The PCR amplification program contained an initial denature at 95°C for 5 min, followed by 25 cycles of denaturing at 95°C for 30 s, annealing at 55°C for 30 s, and extension at 72°C for 30 s, followed by a final extension at 72°C for 5 min. The thermal cycling for archaea was similar to that for bacteria except that the annealing temperature was 57°C. After amplification, the PCR products were purified and quantified, and an equal amount of the PCR product was combined in a single tube to be run on a Roche GS FLX 454 Pyrosequencing machine at Majorbio Bio-Pharm Technology Co., Ltd., Shanghai, China.

### Analysis of Pyrosequencing-derived Data

After sequencing completed, all sequence reads were quality checked using Mothur software [13]. Raw sequence reads were filtered before subsequence analyses to minimize the effect of random sequencing errors. The sequence reads that did not contain the correct primer sequence after the initial quality check (primer sequences were subsequently removed), were shorter than 200 bp, contained one or more ambiguous base(s), or checked as chimeric artifact were eliminated. Finally, the high-quality sequences after filtering were assigned to samples according to barcodes. Sequences were aligned in according with SILVA alignment [14]. Mothur was also used to conduct rarefaction curve, abundance base coverage estimator (ACE), richness (Chao), Shannon diversity, Simpson diversity indices and Good's coverage analysis, assign sequences to operational taxonomic units (OTUs, 97% similarity) using furthest neighbor approach. For taxonomy-based analysis, the SILVA database project (http://www.arb-silva.de) was used as a repository for aligned rRNA sequences. The sequences have been deposited into the NCBI short read archive (SRA) under the accession number SRX484115 for bacteria and SRX485028 for archaea.

### Analytical methods

Volumes of produced biogas were measured by wet gas meters every day. The methane content of the biogas was measured by a gas chromatograph (GC) (Agilent Technologies 6890N, CA, USA) with a thermal conductivity detector equipped with Hayseq Q mesh and Molsieve 5A columns. For the analysis of volatile fatty acid (VFA), the fermentation mixtures withdrawn from digesters were centrifuged at 10, 000 ×g for 10 min, and then the supernatants were immediately filtered through 0.45 um cellulose nitrate membrane fiber paper. The filtrate was collected in a 1.5 ml gas chromatograpgy (GC) vial and acidified by formic acid to adjust the pH to approximately 2.0, and then analyzed using a gas chromatograph (GC, Agilent 7820) with a flame ionization detector (FID) and equipped with a 52 CB column (30 m×0.32 mm×0.25 mm). The concentration of total VFA was calculated as the sum of the measured acetic, propionic, n-butyric iso-butyric, n-valeric, and iso-valeric acids. Metrohm 774 pH-meter was used in all pH measurements. The TS, VS, total alkalinity (TA) and total ammonia-nitrogen (TAN) were measured according to Standard Methods [15]. Free ammonia-nitrogen (FAN) was calculated in the same way as described by Østergaard [16]. The degradation or removal level based on VS (i.e., VS reduction) was calculated by the same formula as reported previously [3]. All experimental analyses were performed in triplicate. The data on performances of each reactor were expressed as mean±standard deviation of the samples.

## Results and Discussion

### Effect of TS content on anaerobic digestion performance

Table 2 summarizes the values of the main parameters indicating system stability (pH, VFA, TA) and potential inhibitory chemicals (TAN and FAN) for three reactors operated at different TS contents, and the performance data were the average values of the last five samples during the operation period of the fifth SRT after the system reached steady state (determined by constant methane yield and VS reduction).

**Table 2:** Summary of performance parameters on system stability and inhibition in three reactors.

							VFA^f^ (g/L)	
reactor	SRT^a^	OLR^b^ (Kg VS m^−3^d^−1^)	pH	TA^c^ (g/L)	TAN^d^ (g/L)	FAN^e^ (mg/L)	Total	Acetic
R1(5%)	20	2.35	7.39±0.08	3.8±0.1	0.40±0.01	11±0.4	0.12±0.01	0.11±0.01
R2(15%)	20	7.01	7.68±0.06	10.9±0.3	1.31±0.15	66±2.5	0.53±0.02	0.43±0.01
R3(20%)	20	9.41	7.82±0.09	13.8±0.2	1.92±0.04	163±8.0	0.94±0.01	0.64±0.02

a SRT: solid retention time.

b OLR: organic loading rate.

c TA: total alkalinity.

d TAN: total ammonia nitrogen.

e FAN: free ammonia nitrogen.

f VFA: volatile fatty acid.

For each semi-continuously experiment with a good anaerobic digestion performance (between 5% and 20% TS), there was no accumulation of VFA and low pH. The concentration values of VFA showed increasing trend with increasing TS contents. Under mesophilic semi-dry anaerobic digestion of sorted organic fraction of municipal solid waste (OFMSW), Li et al., also observed an increasing trend of the VFA concentrations with TS contents increasing (for TS contents of 11.0%, 13.5% and 16.0%), the maximum VFA value was 4.2 g L^−1^, 6.8 g L^−1^ and 22.4 g L^−1^, respectively [17]. In this study, higher VFA concentrations were obtained in the reactors with higher TS contents, which could be explained by the fact that more organic matter was hydrolyzed and transformed to VFA in the reactors. High VFA levels and almost steady VS reduction (Table 3) in reactors indicated that the acidogenic activity was not influenced significantly. In addition, the reactor stability was maintained and the digestion occurred normally because a constant pH was maintained for each reactor. The average pH value was about 7.39, 7.68 and 7.82 at 5%, 15% and 20% TS, respectively. These pH values were within the permissible range for AD 6.5–8.5 but not with the optimal range 6.8–7.4 [18]. As we all know, the increase of VFA concentration contributes to the decrease of pH. However, low pH value was not observed in R3 in which the VFA concentration was highest. It could be explained by the fact that high buffering capacity was observed in high-solids anaerobic system at TS 20%, for which the total alkalinity value of 13.8 g CaCO_3_/L was detected.

**Table 3:** Performance parameters of three reactors with different total solids contents.

Reactor	SRT (d)	OLR (Kg VS m^−3^d^−1^)	Y _biogas_ a (LBiogas gVS^−1^_added_)	CH_4_ (%)	Y_methane_ b (L CH_4_ g VS^−1^_added_)	VS_r_ c (%)	SBP^d^ (L Biogas gVS^−1^_removed_)	SMP^e^ (LCH_4_ gVS^−1^_removed_)	BP^f^ (Biogas L^−1^d^−1^)	MP^g^ (LCH_4_ L^−1^d^−1^)
R1(5%)	20	2.35	0.70±0.02	52.5±2.1	0.37±0.01	80.1±2.4	0.88±0.02	0.46±0.01	1.65±0.06	0.87±0.03
R2(15%)	20	7.01	0.76±0.01	54.2±2.7	0.41±0.01	82.4±2.2	0.92±0.05	0.50±0.01	5.36±0.2	2.90±0.07
R3(20%)	20	9.41	0.87±0.02	55.1±2.6	0.48±0.01	85.6±2.6	1.01±0.04	0.56±0.02	8.21±0.34	4.52±0.05

a Y_biogas_: biogas yield.

b Y_methane_: methane yield.

c VS_r_: VS reduction.

d SBP: specific biogas production rate based on removed VS.

e SMP: specific methane production rate based on removed VS.

f BP: volumetric biogas production rate.

g MP: volumetric methane production rate.

It was known that ammonia nitrogen concentration (especially free ammonia concentration) was an important factor influencing the stability of anaerobic digestion system. The TAN and FAN concentrations in three reactors at steady state were also observed. They showed a similar trend to that of above parameters with increasing TS contents. However, the maximum FAN value was just 163 mg/L. It has been reported that the FAN at concentrations above 200–1100 mg/L can inhibit the anaerobic system [19]. Therefore, the effect of FAN concentration on the system stability was probably negligible for the three reactors with TS contents ranged from 5% to 20%.

Biogas generation and methane efficiency of different reactors are shown in Table 3. The average daily cumulative biogas (based on added VS) of R1-5%, R2-15% and R3-20% accounted to 700, 760 and 870 ml and 370, 410 and 480 ml methane content, respectively. Hence, both of biogas production and methane content showed increasing trend with increasing TS contents. This result was in contrast with a previous work [2], in which the reactors with smaller TS contents showed higher biogas production and methane percentage in the batch anaerobic digestion of FW. It was suggested that the increasing of feeding TS contents lower than 20% has positive effect on the methane production. A maximum methane content of 55.1% in R3 agreed with the previous study on anaerobic digestion of FW [1], but was lower than in another reference [20], which was probably due to the differences in substrate composition. In addition, it could also be observed that reactors with higher TS contents showed higher volumetric biogas and methane production rate. It is well known that FW is a high degradable substrate for anaerobic digestion [21]. For reactors R1-R3 at a fixed 20 days SRT, increased feeding TS content of FW meant higher applied OLR and larger proportion of easily degradable substrate for microorganisms, which results in higher volumetric biogas yield and methane production rate. As showed in Table 3, higher VS reduction was observed in the anaerobic digesters with higher TS contents. The reasons for this important result obtained were investigated from the microbiology aspect in the following chapters. The specific biogas and methane product rate based on removed VS increased slightly. The highest specific biogas production rate determined on removed VS was 1.01 L gVS^−1^ removed in R3, which was higher than corresponding data obtained in a previous study [1].

### Overall analysis of pyrosequencing

The latest developed 454 high-throughput pyrosequencing that can generate huge amounts of DNA reads is widely employed to investigate the bacterial and archaeal community structures and dynamics in various environmental samples. To investigate the compositions of microbial populations involved in the fermentative reactors with different TS contents, a total of 9571, 7769 and 5598 trimmed bacterial 16S rRNA gene sequences and 5245, 4654 and 4432 trimmed archaeal 16S rRNA gene sequences were recovered from samples R1, R2 and R3 (Table S1), respectively. The sequences were grouped into OTUs at a distance level of 3% to estimate the phylogenetic diversities of microbial communities. The OTUs number identified by bacterial sequences in R1 was the largest among three samples. The bacterial community richness levels can also reflected using ACE, Chao, Shannon and Simpson diversity indices (Table S1), which also revealed that the R1-5% had the highest bacterial diversity among three samples. However, the number of archaeal OTUs in R2 was the largest. The rarefaction curves of three samples generated at 3% cutoff for bacterial and archaeal communities are shown in the Figure S1 (Supporting information), demonstrating clearly that the bacterial community richness of R1 and the archaeal community richness of R2 was the highest among these samples, respectively. However, none of the curves approached a plateau, suggesting that this sequencing depth was still not enough to cover the whole microbial diversity and further sequencing would have resulted in more OTUs for each sample. Pyrosequencing analysis of environmental samples can obtain much more sequences and OTUs than conventional cloning and sequencing methods [11], [12]. The bacterial (or archaeal) PCR amplicons from anaerobic digester were grouped into only 238–514 (or 8–26) OTUs according to the clone library in a previous publication [22]. To the authors's knowledge, this was the first study using pyrosequencing technology to characterize the microbial communities in anaerobic digesters with different TS contents. It can be found that compared with traditional clone library, 454 high-throughput pyrosequencing could be a powerful tool to elucidate the microbial community structures and diversities in anaerobic reactors treating food waste with different TS contents.

### Effect of total solids content on functional bacterial populations involved in food waste hydrolysis and acidification

**Figure 1:**
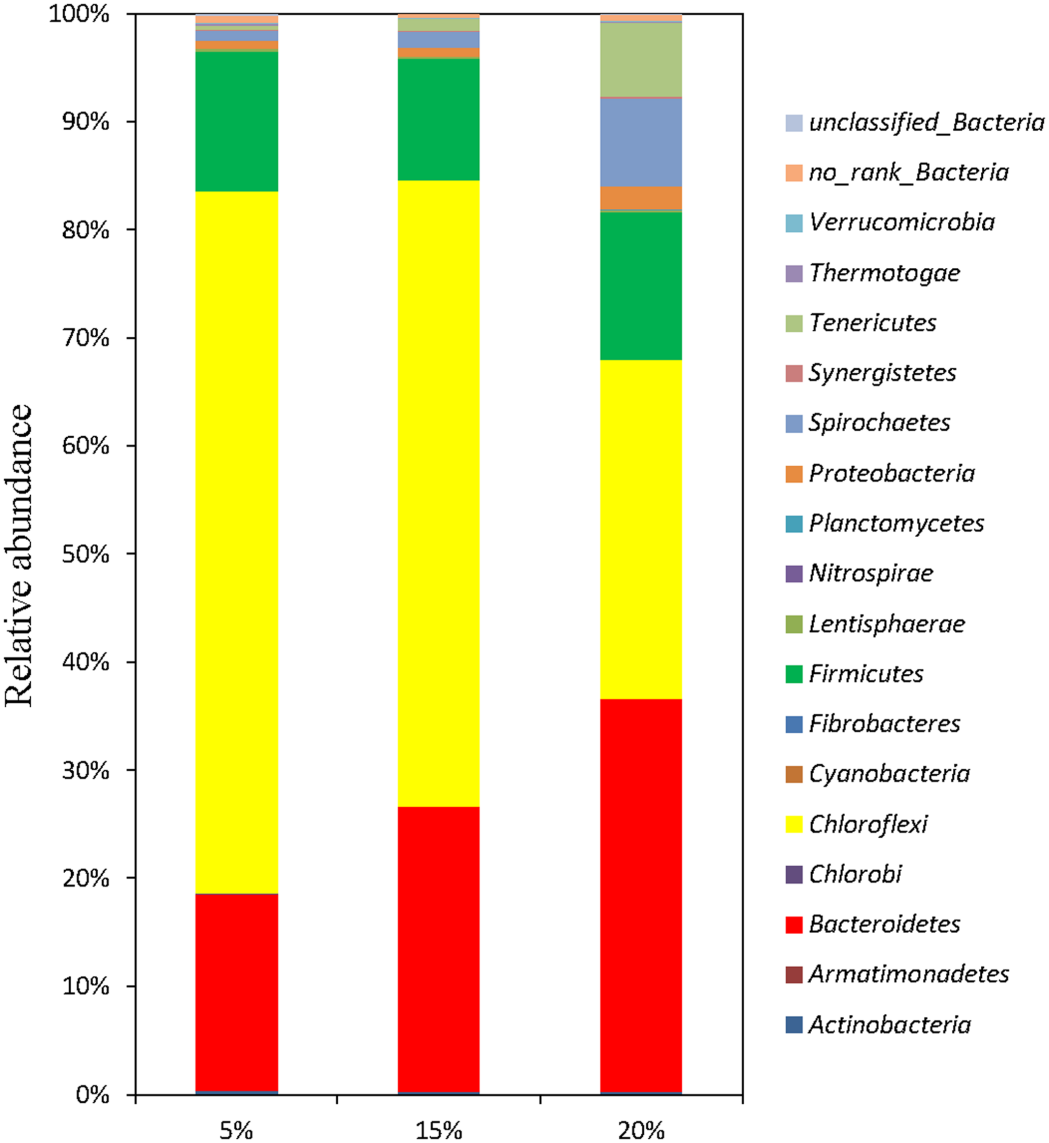
Taxonomic compositions of bacterial communities at phyla level in each sample retrieved from pyrosequencing.

Large numbers of bacterial populations are involved in the hydrolysis and acidification processes of anaerobic fermentation for food waste. The distribution of sequences at the phylum level in each sample is shown in Figure 1. There are seven phyla with relative abundance of higher than 0.5% in at least one sample. From the phylum assignment results, it can be seen that most bacterial sequences in the anaerobic digester treating food waste were distributed among three major phyla: *Chloroflexi*, *Bacteroidetes* and *Firmicutes*, the total relative abundances of them accounted for 96.13%, 95.61% and 81.35% in R1, R2 and R3, respectively, along with other phyla at minor predominance. Similarly, *Bacteroidetes*, *Firmicutes*, *Chloroflexi*, *Synergistetes*, and *Actinobacteria* were reported to the major populations at phylum level in the mesophilic anaerobic digester treating food waste [4]. The dominance of *Bacteroidetes*, *Firmicutes* and *Chloroflexi* was also found in other previous studies [7], [23]. In addition, R3 with 20% feeding TS content had high relative abundance of *Spirochaetes* (8.09%), *Tenericutes* (6.86%) and *Proteobacteria* (2.16%).

Although most bacteria in reactors were affiliated to these dominant phyla, the relative abundances of these phyla in each reactor were different and each digester had its own characteristic bacterial community composition. The proportion of phylum *Chloroflexi* in each reactor was the highest in this study. This was in good accordance with previous reports that *Choroflexi* populations were abundant in anaerobic digesters, as determined by membrane hybridization [7], FISH [23] and 16S rRNA gene clone analysis [23], [24]. Rivière et al., also found large proportions (25–45%) of *Chloroflexi* sequences in municipal WWTP sludge samples [22]. An important trend is the small proportion of *Choroflexi* at the highest TS content: 31% for the 20% TS, compared to 58% with the 15% TS and 65% at the 5% TS. The proliferation of *Choroflexi* (formerly known as Green Nonsulfur Bacteria), a well known scavenger biomass-derived organic carbon such as soluble microbial products (SMP), supports a greater influence of difficult-to-biodegrade organic materials from the input substrates and from endogenous dacay of the anaerobic biomass [22], [25]. For R1-R3 at a fixed SRT, increased feeding TS of FW meant higher applied OLR and larger amount of easily degradable substrate per unit volume for microorganisms, which resulted in a smaller relative abundance of phylum *Choroflexi*.

On the other hand, the *Bacteroidetes* population was enriched in the reactors with higher TS contents (from 18.2% at the 5% TS to 26.40% at the 15% TS and 36.33% at the 20% TS). The phylum *Bacteroidetes* are proteolytic bacteria and were probably involved in the degradation of various proteins used for anaerobic digestion studies [22], [25]. The majority of proteolytic microorganisms are able to metabolize amino acids to produce VFA such as acetate, propionate and succinate and NH_3_ [22]. Interestingly, their selective enrichment at high TS contents seems to be in consistent with the observation of high protein-input rate and VFA production in the reactors with higher TS contents (Table 2). This result indicated the importance of the *Bacteroidetes* performing protein hydrolysis. However, the changing trend of relative abundance of the phylum *Firmicutes* was not obvious with increasing TS contents. The average value of *Firmicutes* proportion was 12% in three reactors. *Firmicutes* are well-known to be acetogenic and syntrophic bacteria that can degrade VFA, such as butyrate and its analogs. The prevalence of organisms belonging to *Firmicutes* suggested that these products are readily available due to the prior fermentation of these simple VFA and played a critical role in anaerobic digestion of FW, especially on the production of acetic acid, an essential step for methane production by acetoclastic methanogenic microorganisms. In addition, the relative abundances of other phyla including *Proteobacteria*, *Spirochaetes* and *Tenericutes* obviously increased with the feeding TS contents increasing. It has been suggested that they might play important roles in the degradation of FW. *Proteobacteria* are also involved in the first step of the degradation of organic wastes and they are important consumers of propionate, butyrate, and acetate [23]. *Spirochaetes* are reported to ferment carbohydrates or amino acids into, mainly, acetate, H_2_ and CO_2_ [8] and *Tenericutes* was found to be related with lignin utilization [26].

In order to further compare the difference of bacterial communities in anaerobic digesters with different feeding TS contents, it is preferable to deconstruct the sequencing date at the subdivision level. Therefore, the relative abundance of each genus in three samples was calculated. The sequence distributions at genus level in each sample are shown in Table 4. A total of 17 genera were detected among which 7 genera with relative abundance of higher than 0.5% in at least one sample were screened as the abundant genera. Other genera were grouped into the minors. As mentioned in the previous section, lower proportions of population from the phylum *Choroflexi* were markedly detected in the reactors with higher TS contents. All sequences classified to phylum *Choroflexi* in three reactors were assigned to genus *Anaerolineaceae* (Figure 1 and Table 4) and class *Anaerolineae* at class level (previous known as “subphylum I” [24]) (Table S2), and the relative abundance of genus *Anaerolineaceae* decreased with increasing TS contents. Because all the characterized species of the class *Anaerolineae* are anaerobic bacteria that decompose carbohydrates via fermentation [27], the genus *Anaerolineaceae* seemed to be involved in carbohydrate decomposition in anaerobic digestion of FW. Similarly, in the previous studies, it was found that all the *Choroflexi* sequences obtained from the up-flow anaerobic sludge blanket reactors treating various food-processing and high-strength organic wastewaters belong to the class *Anaerolineae* [27] and *Anaerolineaceae* group was dominant in phylum *Choroflexi* with its maximum proportion of 8.9% at the 58 days in mesophilic anaerobic digestion of FW [4].

**Table 4:** Taxonomic composition of bacterial communities at the genus level for the sequences retrieved from each sample.

		5%		
Phylum	Genus	Relative abundance 15% 20%		
	*Bacteroidales*	0.43%	0.40%	1.54%
	*Bacteroides*	0.54%	0.27%	0.82%
	*Barnesiella*	2.83%	0.08%	0.00%
	*Marinilabiaceae*	0.37%	0.22%	0.61%
*Bacteroidetes*	*Parabacteroides*	0.18%	0.06%	0.34%
	*Petrimonas*	0.45%	0.37%	1.11%
	*Proteiniphilum*	1.15%	2.59%	4.29%
	*Rikenellaceae*	11.16%	21.70%	26.58%
	*Sphingobacteriales*	0.48%	0.17%	0.52%
*Chloroflexi*	*Anaerolineaceae*	64.99%	58.03%	31.37%
	*Anaerobranca*	0.11%	0.36%	1.43%
	*Christensenellaceae*	0.30%	0.14%	0.18%
	*Clostridiales*	1.73%	4.00%	3.88%
	*Erysipelotrichaceae*	7.17%	1.85%	2.41%
*Firmicutes*	*Fastidiosipila*	0.30%	0.79%	1.09%
	*Gelria*	0.85%	0.85%	0.45%
	*Lachnospiraceae*	0.13%	0.72%	0.39%
	*Lutispora*	0.09%	0.06%	0.36%
	*Ruminococcaceae*	1.11%	0.75%	0.86%
*Proteobacteria*	*Novosphingobium*	0.21%	0.14%	0.77%
	*Rhizobiales*	0.07%	0.06%	0.36%
*Spirochaetes*	*Spirochaeta*	0.28%	0.26%	0.73%
	*Spirochaetes*	0.46%	1.08%	6.98%
*Tenericutes*	*Acholeplasma*	0.40%	1.12%	6.75%
	Minor group	4.21%	3.93%	6.20%

Concerning *Bacteroidetes*, another very abundant phylum which increased with the increasing TS contents, the subdivisions at genus level were multiple and many genera were mainly presented in three anaerobic reactors. *Rikenellaceae* spp. and *Proteiniphilum* spp. were the mostly major genera within this dominant phylum and the changing trends of the relative abundances of these two genera were the same as that of the *Bacteroidetes*. *Rikenellaceae* spp. showed a remarkable proportion from 11% to 27%. The genus *Rikenellaceae* could utilize lactate as substrate in the fermentation processes, and acetate and propionate are the main end-products [28]. *Proteiniphilum*, a relatively new genus showed an unusual ability to grow well at 20–45°C and pH 6.0–9.7. The strains were proteolytic and yeast extract, peptone and l-arginine could be used as carbon and energy sources. Acetic acid and NH_3_ were produced after utilizing these substrates [29]. The predominance of *Proteiniphilum* was also obtained in other anaerobic digesters by using a meta-analysis approach [9]. Other genera in this phylum with individual proportion higher or lower than 0.5% might also have played important roles in FW degradation. Regarding to *Firmicutes*, the generic distributions were also distinct with genera *Clostridiales* and *Erysipelotrichaceae* as the main groups in three anaerobic reactors (Table 4). The latter was especially notable in sample R1-5% with relative abundance of 7.13%. Moreover, the proportion of the reigning genera *Spirochaetes* within the abundant phylum *Spirochaetes* and *Acholeplasma* within the *Tenericutes* increased obviously with TS contents increasing. From the analyses made above, it can be seen that the changing patterns of main microbial population abundances were closely related to the performance variations with TS contents increasing, especially for VS reduction. The increasing degradation of organic matter to precursors for methanogenesis was jointly accomplished by the compatible collaborations of these microorganisms which played their respective roles in one of several trophic levels including hydrolysis, fermentation and acetogenesis.

### Effect of total solids content on functional archaeal populations involved in food waste methanogenesis

**Figure 2:**
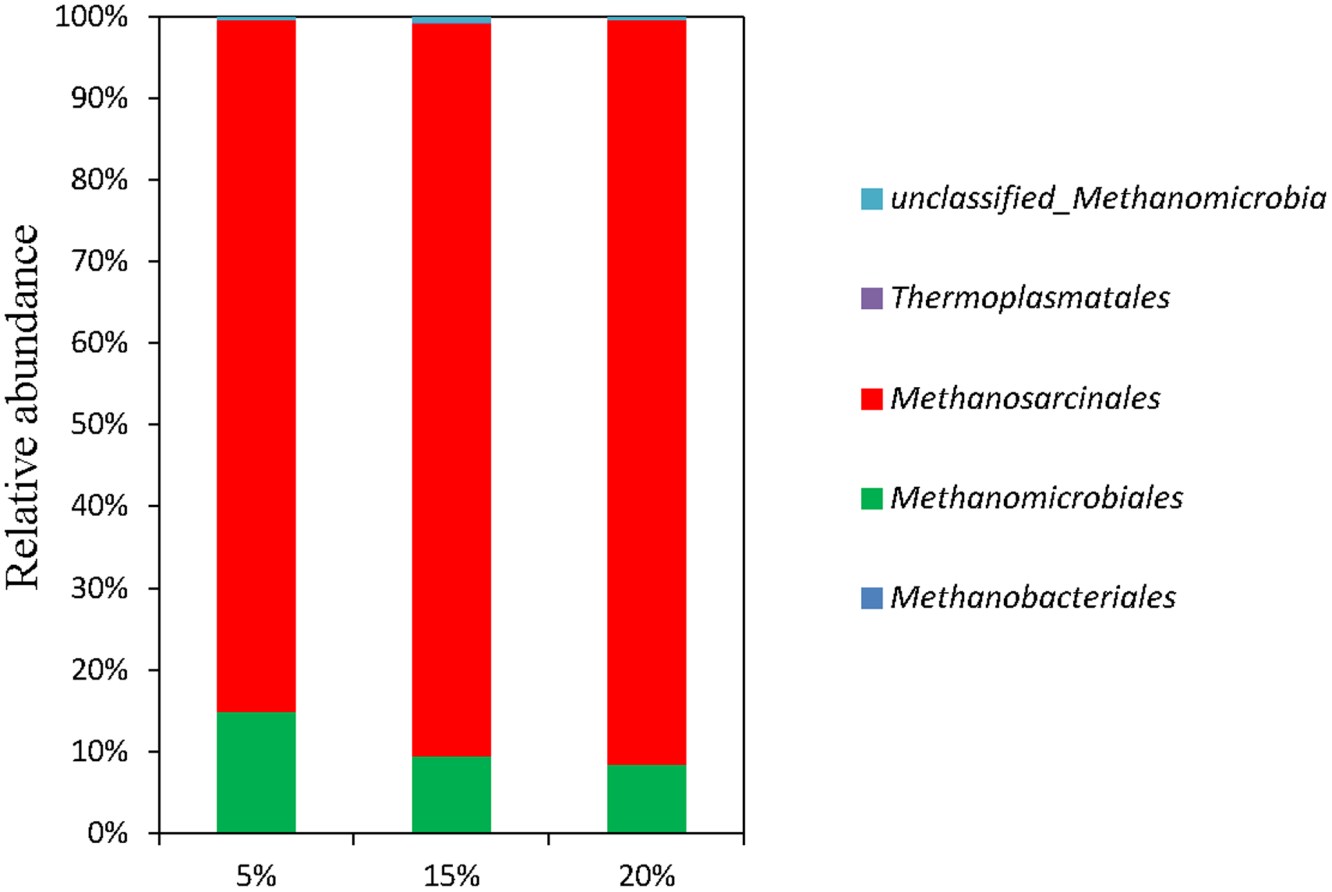
Taxonomic compositions of methanogens at order level in each sample retrieved from pyrosequencing.

The diversities of archaeal populations in three anaerobic digesters were also revealed by high-throughput pyrosequencing target 16S rRNA gene segments. All species richness estimators including ACE, Chao Shannon and Simpson indices are shown in Table S1. The Good's coverage estimated at least 97% coverage at a similarity of 97%, indicating good coverage of archaeal community. Two hydrogen-utilizing methanogenic groups, *Methanobacteriales* and *Methanomicrobiales*, and acetoclastic methanogenic order *Methanosarcinales* were detected in three reactors. The sum relative abundances of these three methanogenic groups accounted for 99.64%, 99.19% and 99.62% of total archaeal sequences in R1, R2, R3, respectively. However, *Methanococcales* was not detected in any DNA samples in this study (Figure 2). This result was in accordance with previous work characterizing the microbial community shifts in anaerobic digestion of secondary sludge [30]. The relative abundances of sequencing data were also analyzed more specifically at genus level (Table. S3). It was showed that the phylogenetic diversity of methanogens was much lower than that of the bacterial community due partly to the inherent phylogenetic low diversity of methanogens.

As shown in Table S3, there was no large gap in terms of methanogens diversity and distinct discrimination in the taxonomic compositions at genus level. Most of methanogens were assigned to the genus *Methanosarcina* (accounting for 84.4%, 89.5% and 90.9% of total archaeal sequences in R1, R2, R3, respectively), indicating that acetoclastic methanogens played important roles in anaerobic digestion of FW and acetoclastic methanogenesis was the principal pathway of methane production. The low-solids anaerobic digester R1 was secondly dominated by hydrogenotrophic *Methanoculleus* while another hydrogenotrophic methanogens *Methanomicrobiales* was the second most detected group in anaerobic digesters R2 and R3.

*Methanosarcina*, a typical member of acetoclastic methanogens, have been often reported as the dominant methanogens in AD [31]. The ability of genus *Methanosarcina* having high growth rates and forming irregular cell clumps makes them more tolerant to changing in pH and high concentrations of toxic ionic agents [32]. The genus *Methanosarcina* produce methane from acetate, although some species are more versatile and can also utilize H_2_/CO_2_, methylated amines and methanol. In addition, *Methanosarcina* spp. are able to use both the acetoclastic and the hydrogenotroph methanogenesis pathways, making them more tolerant to specific inhibitors of the acetoclastic pathway compared to *Methanosaeta* spp. Therefore, anaerobic digester dominantly based on *Methanosaricna* spp. could potentially achieve stable methanogenesis [33], as their special morphological characteristics and flexibility in metabolism.

Besides, the changing patterns of the proportions of three major genera with TS contents increasing were different. The relative proportion of the genus *Methanosaricna* slightly increased from 84% to 90.9% with the TS content increased from 5% in R1 to 20% in R3. On the basis of stable operation, increased feeding TS contents of FW meant higher applied OLR and more VS for microorganisms, which resulted in higher VFA concentrations. In this study, it was observed in Table 2 that the acetate concentration increased with increasing TS contents. It is suggested that higher acetate concentrations would favor the growth of *Methanosarcina* [33]. Therefore, higher concentrations of VFA (especially acetate) and, by extension, at higher OLR caused by the anaerobic systems with higher TS contents induced the selective proliferation of *Methanosarcina*.

The relative abundance of genus *Methanoculleus* obvious decreased from 7.63 to 2.91% with increasing TS contents, indicating that hydrogenotrophic methanogenesis by *Methanoculleus* contributed less to the methane production in high-solids AD than it did in low-solids AD. It has been reported that *Methanoculleus* methanogens had been widely distributed with large proportion in various thermophilic ananerobic digesters [34] and their population ratio seems to be affected by HRT, OLR, or the concentration of VFA. In this study, similar result was obtained that the dominance of *Methanoculleus* declined in the mesophilic anaerobic digesters with TS content increasing resulting in the increase of OLR and the concentration of VFA. Summarily, the changing of microbial communities in mesophilic anaerobic digestion of FW was responsible for the different performances of the reactors with the increasing TS contents. The results obtained in this study expand our knowledge about the role of the TS content on the behavior of the microbial community structure involved in the anaerobic digestion degradation of solids, from low-solids to high-solids technology, and hence to provide valuable information to optimize fermentation process to favor efficient breakdown of food waste.

### Conclusions

Three stable processes were achieved for AD of food waste with TS contents increasing from 5% to 20%. Better performances, mainly including VS reduction and methane yield and significant shifts in bacterial community, were obtained with the increasing TS contents. The relative abundance of phylum *Chloroflexi* decreased while other functional bacteria increased. The genus *Methanosarcina* absolutely dominated in archaeal communities in three reactors and the relative abundance of this group showed increasing trend with TS contents increasing. These results revealed the effect of the TS content on the performance parameters and the behavior of the microbial community involved in the AD of food waste from wet to dry technologies.

## Supporting Information

Figure S1 **Rarefaction cures of bacterial (A) and archaeal (B) sequences from the fermentation reactors with different total solids contents.**
(DOCX)

Table S1 **Bacterial and archaeal richness and diversity indices for three reactors.** All values were calculated at a distance level of 3%.
(DOCX)

Table S2 **Taxonomic composition of bacterial communities at the class level for the sequences retrieved from each samples.**
(DOCX)

Table S3 **Taxonomic composition of archaeal communities at the genus level for the sequences retrieved from each samples.**
(DOCX)
