# Supplementary material for: Effect of Increasing Total Solids Contents on Anaerobic Digestion of Food Waste under Mesophilic Conditions: Performance and Microbial Characteristics Analysis
Source: PLoS One. 2014 Jul 22;9(7):e102548. doi: 10.1371/journal.pone.0102548 (PMC4106828; doi:10.1371/journal.pone.0102548)

Figure S1. Rarefaction cures of bacterial (A) and archaeal (B) sequences from the fermentation reactors with different total solids contents.


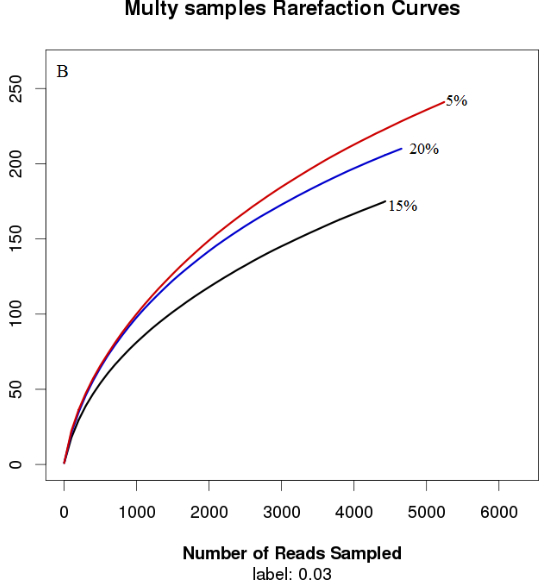

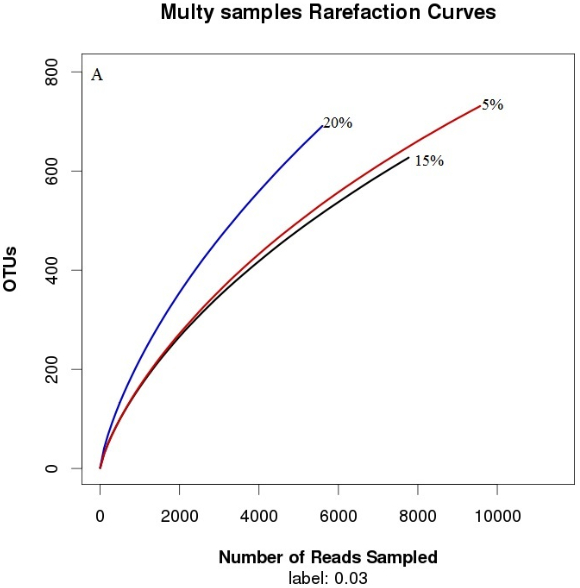

Supplement: Figure S1 — Rarefaction cures of bacterial (A) and archaeal (B) sequences from the fermentation reactors with different total solids contents. (DOCX) [file pone.0102548.s001.docx]
