# Supplementary material for: Effect of Increasing Total Solids Contents on Anaerobic Digestion of Food Waste under Mesophilic Conditions: Performance and Microbial Characteristics Analysis
Source: PLoS One. 2014 Jul 22;9(7):e102548. doi: 10.1371/journal.pone.0102548 (PMC4106828; doi:10.1371/journal.pone.0102548)
Supplement: Table S1 — Bacterial and archaeal richness and diversity indices for three reactors. All values were calculated at a distance level of 3%. (DOCX) [file pone.0102548.s002.docx]

Table S1 Bacterial and archaeal richness and diversity indices for three reactors. All values were calculated at a distance level of 3%.

| Sample  name | Bacteria | | | | | | | Archaea | | | | | | |
| --- | --- | --- | --- | --- | --- | --- | --- | --- | --- | --- | --- | --- | --- | --- |
|  | Effective | OTU | Ace | Chao | Shannon | Simpson | Coverage | Effective | OTU | Ace | Chao | Shannon | Simpson | Coverage |
| R1-5% | 9571 | 731 | 2513 | 1666 | 3.82 | 0.10 | 0.96 | 5245 | 241 | 373 | 295 | 1.75 | 0.50 | 0.98 |
| R2-15% | 7769 | 627 | 1905 | 1313 | 2.72 | 0.33 | 0.95 | 4654 | 378 | 378 | 400 | 1.92 | 0.47 | 0.98 |
| R3-20% | 5598 | 691 | 1928 | 1380 | 2.8 | 0.27 | 0.92 | 4432 | 175 | 370 | 291 | 1.63 | 0.52 | 0.98 |
