# Supplementary material for: Effect of Increasing Total Solids Contents on Anaerobic Digestion of Food Waste under Mesophilic Conditions: Performance and Microbial Characteristics Analysis
Source: PLoS One. 2014 Jul 22;9(7):e102548. doi: 10.1371/journal.pone.0102548 (PMC4106828; doi:10.1371/journal.pone.0102548)
Supplement: Table S2 — Taxonomic composition of bacterial communities at the class level for the sequences retrieved from each samples. (DOCX) [file pone.0102548.s003.docx]

Table S2 Taxonomic composition of bacterial communities at the class level for the sequences retrieved from each samples.

| Phylum | Class | 5% | 15% | 20% |
| --- | --- | --- | --- | --- |
|  |  | Relative abundance | | |
| *Actinobacteria* | *Actinobacteria* | 0.33% | 0.22% | 0.29% |
| *Bacteroidetes* | *Bacteroidia* | 17.52% | 26.22% | 35.71% |
|  | *Sphingobacteriia* | 0.49% | 0.17% | 0.55% |
| *Chloroflexi* | *Anaerolineae* | 64.99% | 58.03% | 31.37% |
|  | *Bacilli* | 0.14% | 0.05% | 0.98% |
| *Firmicutes* | *Clostridia* | 5.62% | 9.28% | 10.22% |
|  | *Erysipelotrichi* | 7.17% | 1.85% | 2.45% |
| *Lentisphaerae* | *Lentisphaeria* | 0.30% | 0.21% | 0.23% |
| *Proteobacteria* | *Alphaproteobacteria* | 0.44% | 0.49% | 1.80% |
|  | *Betaproteobacteria* | 0.10% | 0.18% | 0.30% |
| *Spirochaetes* | *Spirochaetes* | 0.97% | 1.44% | 8.09% |
| *Tenericutes* | *Mollicutes* | 0.40% | 1.17% | 6.86% |
|  | Minor group | 1.53% | 0.69% | 1.15% |
