# Supplementary material for: Effect of Increasing Total Solids Contents on Anaerobic Digestion of Food Waste under Mesophilic Conditions: Performance and Microbial Characteristics Analysis
Source: PLoS One. 2014 Jul 22;9(7):e102548. doi: 10.1371/journal.pone.0102548 (PMC4106828; doi:10.1371/journal.pone.0102548)
Supplement: Table S3 — Taxonomic composition of archaeal communities at the genus level for the sequences retrieved from each samples. (DOCX) [file pone.0102548.s004.docx]

Table S3 Taxonomic composition of archaeal communities at the genus level for the sequences retrieved from each samples.

| Phylum | Genus | 5% | 15% | 20% |
| --- | --- | --- | --- | --- |
|  |  | Relative abundance | | |
| *Methanobacteriales* | *Methanobrevibacter* | 0.02% | 0.00% | 0.00% |
|  | *Methanobacteriaceae* | 0.06% | 0.00% | 0.00% |
| *Methanomicrobiales* | *Methanoculleus* | 7.63% | 1.46% | 2.91% |
|  | *Methanolinea* | 0.02% | 0.00% | 0.00% |
|  | *Methanospirillum* | 1.30% | 1.48% | 0.79% |
|  | *Methanomicrobiales* | 5.98% | 6.55% | 4.81% |
|  | *Methanomicrobia* | 0.25% | 0.67% | 0.27% |
| *Methanosarcinales* | *Methanosaeta* | 0.13% | 0.06% | 0.07% |
|  | *Methanosarcina* | 84.44% | 89.48% | 90.93% |
|  | *Methanosarcinaceae* | 0.04% | 0.15% | 0.09% |
| *Thermoplasmatales* | *Methanosarcinales* | 0.02% | 0.00% | 0.02% |
|  | *Thermoplasmatales* | 0.11% | 0.15% | 0.11% |
